# Author Correction: Protectin DX increases alveolar fluid clearance in rats with lipopolysaccharide-induced acute lung injury

**DOI:** 10.1038/s12276-025-01555-5

**Published:** 2025-09-29

**Authors:** Xiao-Jun Zhuo, Yu Hao, Fei Cao, Song-Fan Yan, Hui Li, Qian Wang, Bi-Huan Cheng, Bin-Yu Ying, Fang Gao Smith, Sheng-Wei Jin

**Affiliations:** 1 Department of Anesthesia and Critical Care, The Second Affiliated Hospital and Yuying Children’s Hospital of Wenzhou Medical University, 325027 Zhejiang, China; 2 Institute of Inflammation and Aging, College of Medical and Dental Sciences, University of Birmingham, Birmingham, UK; 3 Academic Department of Anesthesia, Critical Care, Pain and Resuscitation, Birmingham Heartlands Hospital, Heart of England NHS Foundation Trust, Birmingham, B9 5SS UK

Correction to: *Experimental & Molecular Medicine* 10.1038/s12276-018-0075-4, published online 27 April 2018

After online publication of this article, the authors noticed errors in Figs. 2 and 6. These errors occurred in the preparation and revision of figures, but do not affect the results and conclusions in this study. The authors have provided new versions of Figs. 2 and 6.

Corrected Fig. 2. (PDX group)
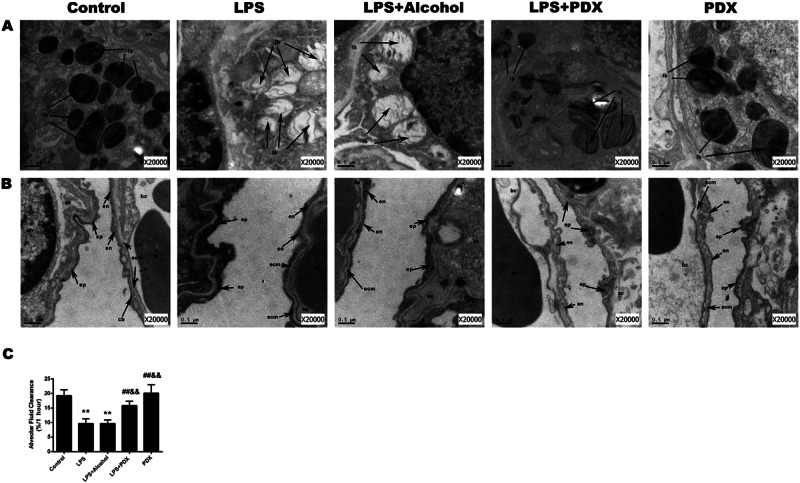


Corrected Fig. 6. (Control group and LPS+Alcohol group)
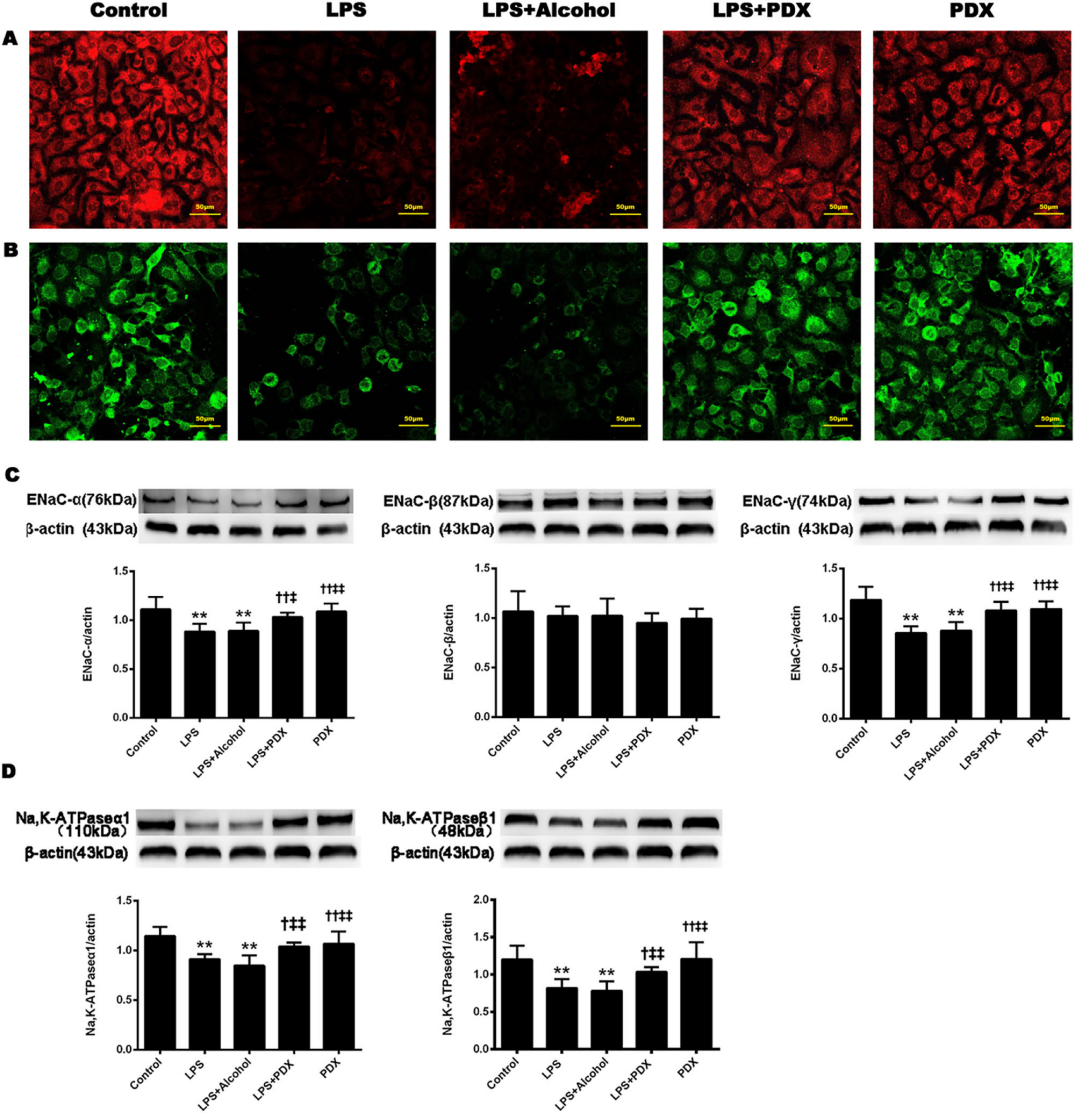


The authors apologize for any inconvenience caused.

The original article has been corrected.

